# Proton Magnetic Resonance Spectroscopy for Diagnosis of Non-Motor Symptoms in Parkinson's Disease

**DOI:** 10.3389/fneur.2022.594711

**Published:** 2022-02-28

**Authors:** Ji-tian Guan, Xin Zheng, Lingfeng Lai, Shuyi Sun, Yiqun Geng, Xiaolei Zhang, Teng Zhou, Huan-ze Wu, Jia-qing Chen, Zhong-xian Yang, Xiao-hong zheng, Jia-xu Wang, Wei Chen, You-qiao Zhang

**Affiliations:** ^1^Department of Radiology, Second Affiliated Hospital of Shantou University Medical College, Shantou, China; ^2^Cell Therapy Center, Beijing Institute of Geriatrics, Xuanwu Hospital Capital Medical University, National Clinical Research Center for Geriatric Diseases and Key Laboratory of Neurodegenerative Diseases, Ministry of Education, Beijing, China; ^3^Laboratory of Molecular Pathology, Shantou University Medical College, Shantou, China; ^4^Department of Computer Science, Shantou University, Shantou, China; ^5^Department of Neurology, Second Affiliated Hospital of Shantou University Medical College, Shantou, China

**Keywords:** early diagnosis, biomarker, Parkinson's disease, proton magnetic resonance spectroscopy, MRI

## Abstract

**Background:**

The current diagnosis of Parkinson's disease (PD) is mainly based on the typical clinical manifestations. However, 60% dopaminergic neurons have died when the typical clinical manifestations occur. Predictive neurobiomarkers may help identify those PD patients having non-motor disorders or in different stage and achieving the aim of early diagnosis. Up to date, few if any neuroimaging techniques have been described useful for non-movement disorders diagnosis in PD patients. Here, we investigated the alteration of metabolites in PD patients in different stage of PD and non-motor symptoms including sleep, gastrointestinal and cognitive dysfunction, by using the 1H-MRS.

**Methods:**

A total of 48 subjects were included between 2017 and 2019: 37 PD (15 men, age 47–82 years) and 11 healthy people (8 men, age 49–74 years). All participants underwent MRI and multi-voxel ^1^H-MRS examination within 3 days in admission. Six kinds of metabolites, such as creatine (Cr), N-acetyl aspartate/creatine (NAA/Cr), N-acetyl aspartate/choline (NAA/Cho), choline/creatine (Cho/Cr), lipid/creatine (LL/Cr), and myo-Inositol/creatine ratio (mI/Cr) were tested among the PD group and the control groups. Statistical analyses and correlation analyses were performed by using SPSS. The *p* < 0.05 was considered statistically significant.

**Results:**

Compared late PD group with a control group or early group, higher Cr ratio and lower NAA/Cr ratio were observed in the late PD group (*p* < 0.05). The mI/Cr in the late PD group was also lower than that in the early PD group (*p* < 0.05). Regarding the relationship between metabolites and NMS, Cho/Cr was higher in the sleep disorder group, whereas mI/Cr was lower in the gastrointestinal dysfunction group in comparison with the non-symptom groups. Moreover, Cr, Cho/Cr, mI/Cr, and LL/Cr were identified to have higher concentrations in the cognitive group in thalamus.

**Conclusions:**

Proton magnetic resonance spectroscopy is an advanced tool to quantify the metabolic changes in PD. Three biomarkers (Cr, NAA/Cr, and mI/Cr) were detected in the late stage of PD, suggesting that these markers might be potential to imply the progression of PD. In addition, subgroups analysis showed that MRS of thalamus is a sensitive region for the detection of cognitive decline in PD, and the alteration of neurochemicals (involving Cr, Cho, mI, and LL) may be promising biomarkers to predict cognitive decline in PD.

## Introduction

With an estimated prevalence of 1–2 case per 1,000 persons above 60 years, Parkinson's disease (PD) is the second common neurodegenerative disease after Alzheimer's disease (AD) (1). Clinical symptoms of PD include motor disorders (such as, quiescent tremor, motor retardation, muscular rigidity, and postural gait abnormalities) (2) and non-motor disorders (sleep disturbances, cognitive disorders, emotional disorders, olfactory impairment, gastrointestinal dysfunction, bladder dysfunction, etc.) (3). The most important pathological change is the degeneration of dopamine (DA) neurons in the substantia nigra where it has been identified to have an accumulation of intracytoplasmic inclusions called Lewy bodies containing α-synuclein (4). Aggregates of α-synuclein have been found to spread from peripheral regions (olfactory bulb and gastrointestinal tract) to the lower brainstem, resulting in early emergence of premotor and non-motor symptoms (NMS) (5). A total of 60% dopaminergic neurons have died when the typical clinical manifestations occur (6). The Hoehn and Yahr scale (H&Y) is commonly used to assess the disease progression (Stage I–II for the early stage of PD, and Stage III–IV for the progressive PD) (7). The Movement Disorder Society–Unified Parkinson's Disease Rating Scale (MDS–UPDRS) and the NMS Scale (NMSS) are, respectively, used to evaluate the clinical severity of motor symptoms (8) and NMS for PD (9). The score of “Recall/Memory” in Mini-Mental State Examination (MMSE) is used to identify the cognitive state in patients with PD (10). However, a lack of objective data may result in decreased diagnostic accuracy of clinical NMS. In addition, early diagnosis before the onset of motor symptoms remains challenging since lack of distinguishable clinical and laboratory markers in premotor PD. Given the background, finding sensitive biomarkers in NMS and early stage PD *via* neuroimaging may not only help understand PD pathogenesis and progression, but also provide better early diagnosis and therapies for patients with PD (11).

Magnetic resonance spectroscopy (MRS) is a MRI technique that allows for direct quantifying microstructural, biochemical, and functional changes in human brain (12). Proton MRS (^1^H-MRS) has been utilized to detect a wide range of endogenous neurochemicals in PD, such as N-acetylaspartate (NAA) as a marker reflecting the density of healthy neurons and the activity of neuronal function, Creatine (Cr) as a marker of energy metabolism, Choline (Cho) as a marker of both membrane catabolism and anabolism, and myo-Inositol (mI) as a marker of glial reaction. Besides, some magnetic resonance peaks overlap between lipid (Lip) and lactic acid (Lac), so the term “LL” is used in this study to qualify the sum of these two substances and to subsequently indicate the ischemia and the hypoxia in the neurodegenerative regions. Whole-brain MRS imaging was performed and the regions associated with NMS were selected for the spectral analysis, such as substantia nigra, pallidum, thalamus, prefrontal cortex, hippocampus, and parahippocampal gyrus (13).

In the previous study, we demonstrated that ^1^H-MRS is a sensitive tool that is able to detect the alterations of neural-related metabolites in multiple cerebral regions, such as lower ratios of NAA/Cr and NAA/Cho in substantia nigra, globus pallidus, prefrontal lobe, hippocampus, cuneus gyrus, and dorsal thalamus (14). In addition, the reduced NAA/Cr and NAA/Cho were positively correlated with the unilateral disorder and cognitive impairment, while negatively correlated with UPDRS score. In the present study, we recollected data and mainly focused on assessing the cerebellar biochemical profile in different stages of patients with PD, especially patients with NMS, by using the ^1^H-MRS. Correlation analysis will subsequently be conducted to investigate the relationship between metabolism levels and the H&Y stage, UPDRS scale, NMSS scale, MMSE score, or clinical factors in patients with PD.

## Materials and Methods

### Subjects

This study was approved by the Ethics Committee of the Second Affiliated Hospital of Shantou University Medical College and was performed according to the Declaration of Helsinki. Written informed consent was obtained from all participants. A total of 48 subjects were included in our hospital between 2017 and 2019: 37 PD (15 mens, age 47–82 years) and 11 health people (8 mens, age 49–74 years). All subjects complied with the diagnostic criteria (2) and diagnosed by a neurologist. Exclusion criteria include (1) history of brain disease (cerebral infarction or cerebral hemorrhage), (2) presence of mental illness or metabolic syndrome, (3) severe heart, lung, liver, kidney, and other organ dysfunction, and (4) inability to provide consent. Patients were evaluated by the modified Hoehn-Yahr stage, UPDRS, NMSS, and MMSE scales. The Hoehn-Yahr stage included stages 1–5, ≤ 1.5 defined as the early stage of PD, while ≥ 1.5 defined as late-stage PD. All participants underwent MRI and multi-voxel ^1^H-MRS examination within 3 days of admission.

### MR Imaging and ^1^H-MRS

**Figure 1:**
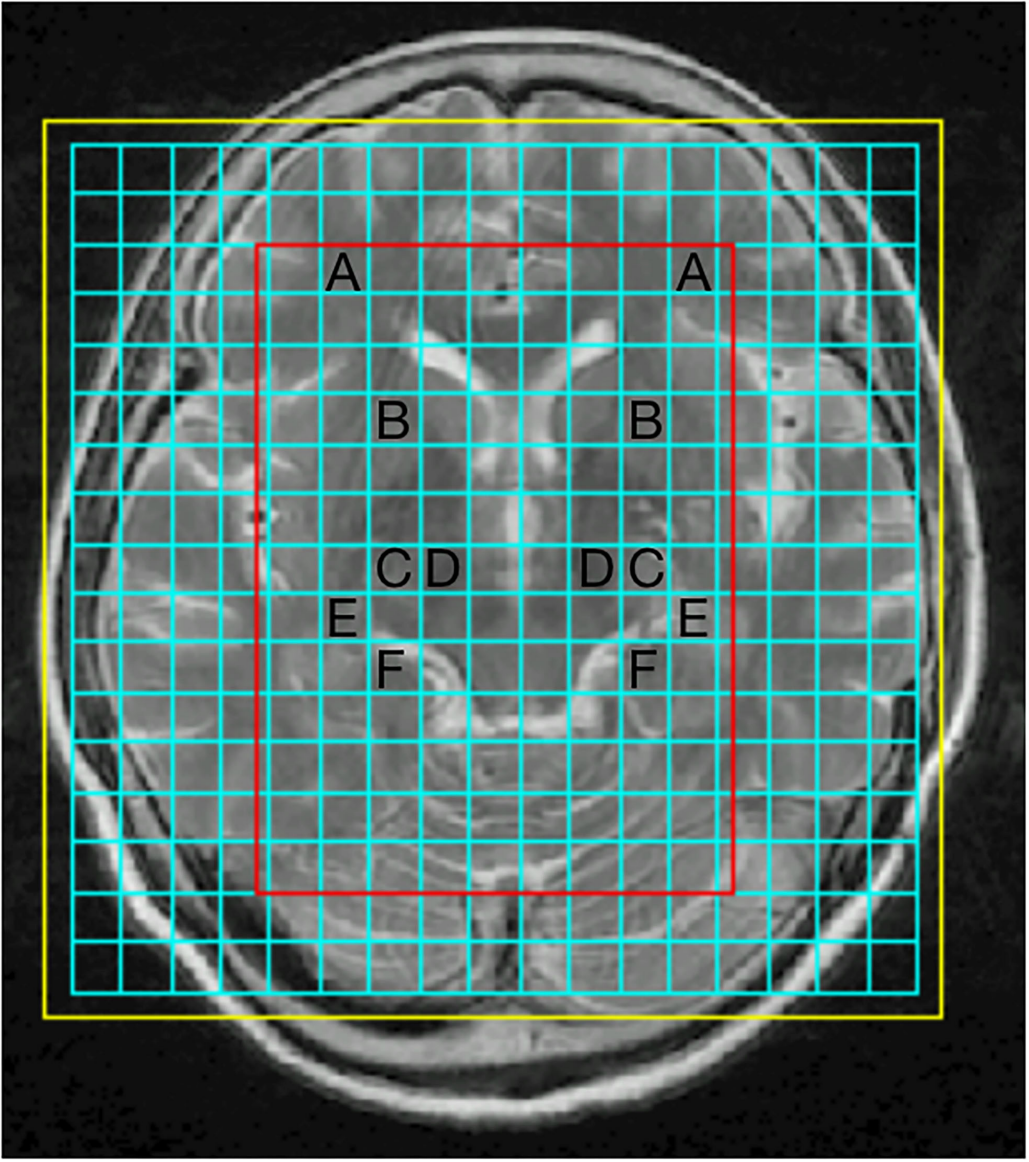
Representative proton magnetic resonance spectroscopy (^1^H-MRS) spectra for four brain regions: prefrontal cortex **(A)**, pallidum **(B)**, thalamus **(C)**, substantia nigra **(D)**, hippocampus **(E)**, parahippocampal gyrus **(F)**.

The 3-T MRI system was used for conventional MRI and ^1^H-MRS (GE signa excite 3T HD Ecospeed) with a standard 8-channel head coil, MR ADW 4.3 workstation Functool software. The axial scanning direction was parallel to the anterior cranial fossa, with a thickness of 5 mm, an interval of 1.5 mm, and a field of vision (FOV) of 24 cm. A total of 20 MRI images were obtained by scanning downward from the cranial top. Scanning parameters of each sequence are as follows: (1) T1WI: Using T1-weighted flux-Attenuated Inversion recovery imaging (T1-FLAIR), repetition time (TR) 2,000 ms, echo time (TE) 24 ms, number of excitations (NEX) = 2, FOV 24 × 18 cm, Matrix 320 × 224, slice thickness 5 mm and interlayer thickness 1.5 mm. (2) T2WI: Using T2-Weighted fast Recovery fast spin-echo imaging (T2-FRfSE), TR 4,600 ms, TE 106 ms, NEX 1.5, FOV 24 × 24 cm, Matrix 384 × 384, slice thickness 5 mm and interlayer thickness 1.5 mm. Multi-voxel ^1^H-MRS was performed using a 2D point resolved spectroscopy (2D PROBE-CSI PRESS) with the following parameters: TR, 1,500 ms; TE, 35 ms; phase × frequency = 18 × 18, volume of interest, 8 × 10 × 1 cm; FOV, 240 × 240 mm and NEX, 1. Voxel regions of interest (ROI) is located in the substantia nigra, pallidum, thalamus, prefrontal cortex, hippocampus, and parahippocampal gyrus. Typical ROI were chosen from the examined brain regions (Figure 1).

**Figure 2:**
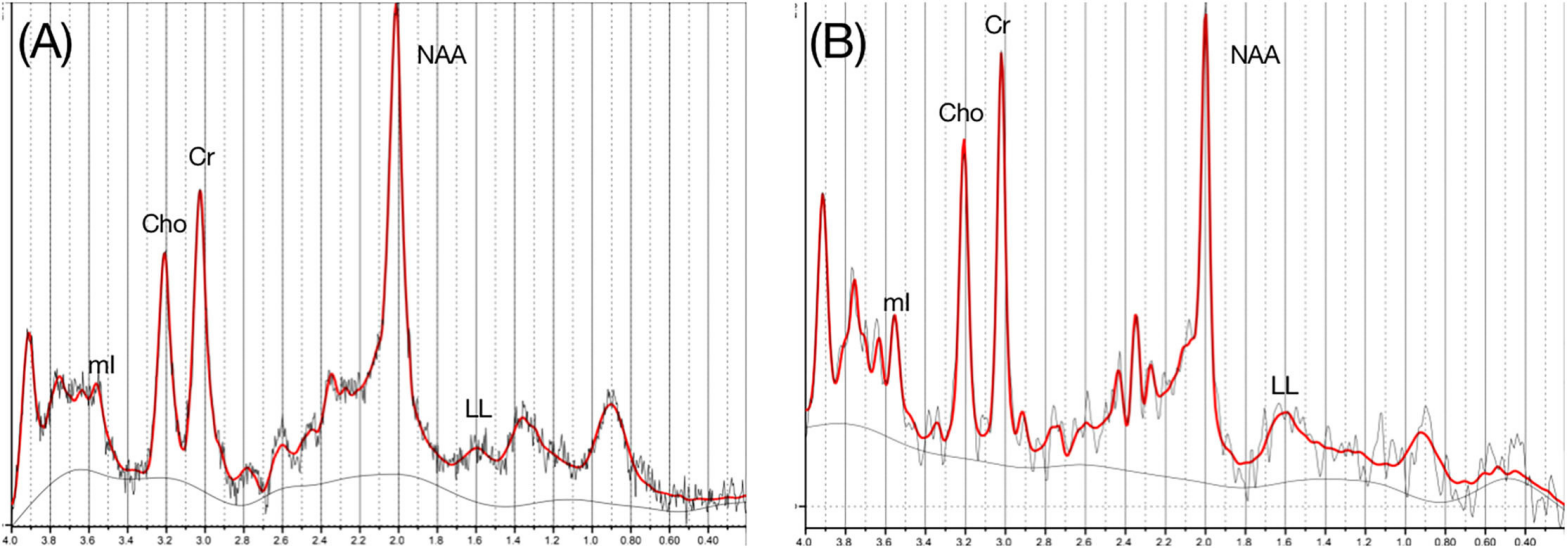
^1^H-MRS spectra obtained from a control subject **(A)** and a patient with PD **(B)**.

The spectral data obtained by GE MR imaging system were analyzed by using Cr as internal reference to calculate the neurometabolites ratio. Original data were sent to ADW4.3 workstation (GE Mediacel System Advantage Window 4.3), and the Functool software was used to process the ^1^H-MRS image. Six kinds of metabolites, such as creatine (Cr), N-acetyl aspartate-to-creatine (NAA/Cr), N-acetylaspartate-to-choline (NAA/Cho), choline-to-creatine (Cho/Cr), lipid-to-creatine (LL/Cr), and myo-Inositol-to-creatine (mI/Cr) ratios were tested among PD group and the control groups. Examples of spectra are presented in Figure 2.

### Statistical Analysis

The cerebral metabolites (Cr, NAA/Cr, NAA/Cho, Cho/Cr, LL/Cr, and mI/Cr ratios) of the early PD group, the progressive PD group and the control group were tested by using SPSS26.0 statistical analysis software. According to the homogeneity of variance, two independent sample *t*-test or approximate *t*-test (*t*-test) were used to compare the cerebral metabolites among the NMS subgroups in the case group. Spearman's correlation analysis was used to analyze the correlation of cerebral metabolites with gender, age, H&Y stage, MDS-UPDRS, NMSS, MMSE scales, hypertension, and diabetes in patients with PD. The *p* < 0.05 was considered statistically significant.

## Results

### Compare Age and Score of H&Y, MDS-UPDRS, NMSS Between Control Group, Early PD Group, and Late PD Group

The 24 cases (H&Y: 1.29 ± 0.46) showed premotor symptoms and 13 cases (H&Y: 3.15 ± 0.38) showed progressive symptoms. Three groups had statistical difference in Age, H&Y score, MDS-UPDRS score, and NMSS score (all *p* < 0.05). The baseline characteristics of the cases are showed in Table 1.

**Table 1:** Demographics and clinical characteristics of the PD patients.

	**Control group (*n* = 11)**	**Early PD group (*n* = 24)**	**Late PD group (*n* = 13)**	***p1***	***p2***	***p3***
Age	57.45 ± 10.09	64.50 ± 10.54	65.84 ± 7.53	0.072	0.029	0.687
H-Y Score	0 ± 0	1.29 ± 0.46	3.15 ± 0.38	0.000	0.000	0.000
MDS-UPDR Score	0 ± 0	28.29 ± 12.82	51.46 ± 18.94	0.000	0.000	0.000
NMSS score	0 ± 0	63.83 ± 14.65	80.23 ± 16.59	0.000	0.000	0.004

*p1 represents the p-value of the control group and the early stage group, p2 represents the p-value of the control group and the progressive group, and p3 represents the p-value of the early stage group and the progressive group*.

### Compare Metabolic Status in Multiple Cerebral Regions Between Control Group, Early PD Group, and Late PD Group

There were statistically significant differences between two compared group (late PD group and control group; late PD group and early PD group) in metabolite ratios, while no statistically differences between early PD group and control group (*p* > 0.05). Due to the limit on the scope of this article, we predominantly presented the positive MRS results in Table 2. Detail metabolic information of MRS results will be summarized in the Supplementary Table 1.

**Table 2:** Summary of the positive MRS results shown in left/right cerebral hemisphere in the early, late PD group and the control group.

		**Early PD group/control group**	**Late PD group/control group**	**Late PD group/early PD group**
Substantia Nigra	Left	–	NAA/Cr ↓	–
	Right	–	NAA/Cr ↓	–
Pallidum	Left	–	–	Cr ↑
	Right	–	–	–
Thalamus	Left	–	–	Cr ↑; mI/Cr ↓
	Right	–	–	–
Prefrontal Cortex	Left	–	–	–
	Right	–	Cr ↑	–
Parahippocampal Gyrus	Left	–	–	–
	Right	–	–	NAA/Cr ↓

**Figure 3:**
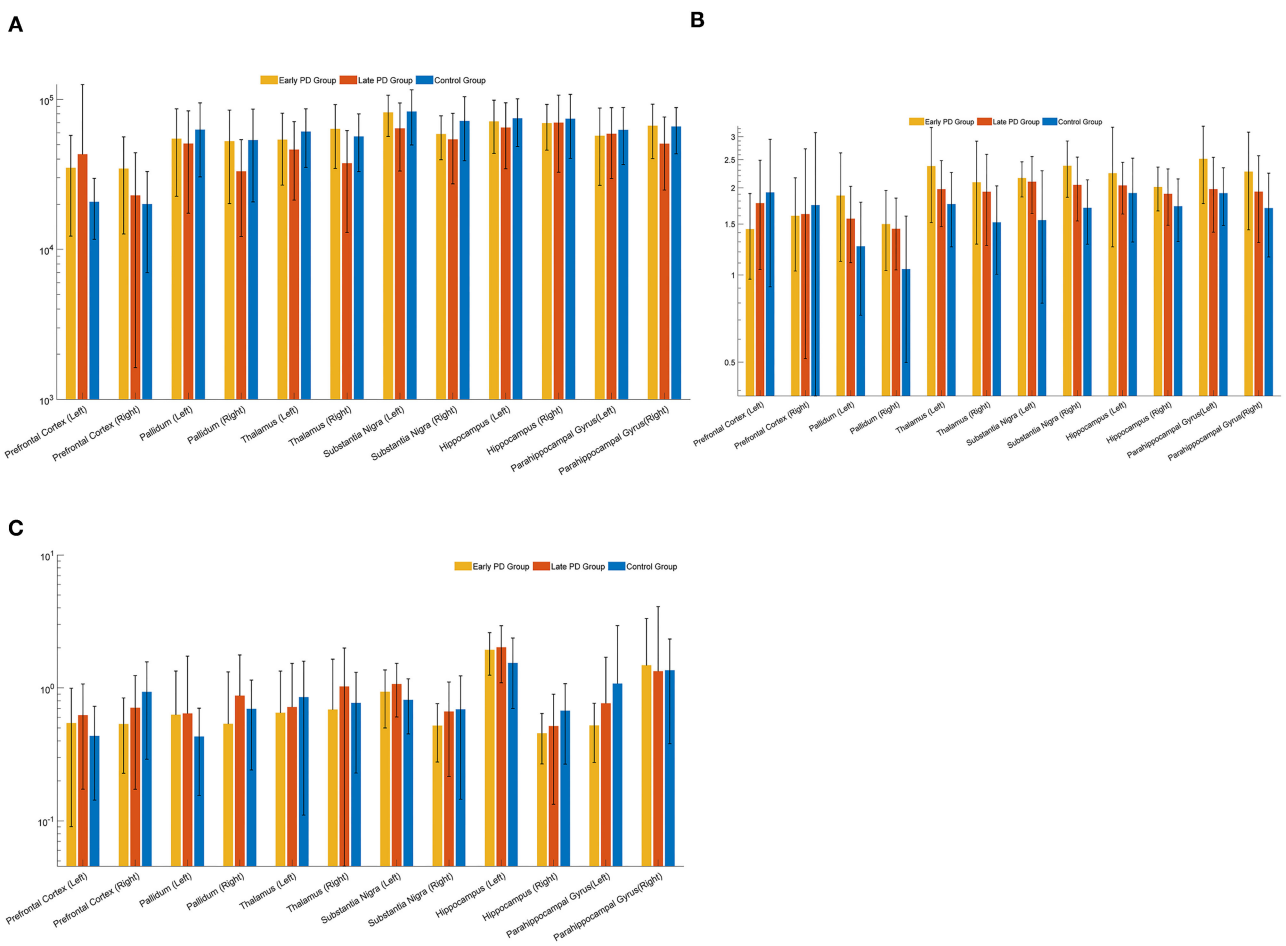
Error bars of mean and standard deviation values of creatine (Cr) **(A)**, N-acetyl aspartate-to-creatine (NAA/Cr) **(B)**, and myo-Inositol-to-creatine (mI/Cr) **(C)**, at different sites in the early, late PD group, and the control group.

Compared late PD group with the control group, Cr in the right prefrontal cortex in the late PD group was higher than that in the control group (*p* < 0.05). NAA/Cr ratio in the bilateral substantia nigra in the late PD group was lower than that in the control group (*p* < 0.05). Compare late PD group with early group, Cr in the left pallium and the left thalamic in the late PD group was higher than that in the early PD group (*p* < 0.05). NAA/Cr in the right parahippocampal gyrus in the late PD group was lower than that in the early PD group (*p* < 0.05). The mI/Cr in the left thalamic in the late PD group was lower than that in the early PD group (*p* < 0.05). Cho/Cr and NAA/Cho were almost no statistically differences between the early PD group, the late PD group, and the control group (*p* > 0.05). Error bars of mean and standard deviation values of Cr, NAA/Cr were shown in Figure 3.

### The Number and Ratio of NMS in PD Group

Overall, 37 patients (24 for early stage PD, 13 for progressive PD) and 11 controls were recruited in the study. For the cases, 70% patients suffering with sleep disorders, nearly 67% patients have gastrointestinal and/or bladder dysfunction, 35% for memory decline (shown in Table 3). The NMS gradually increase and worsen as PD disease progresses.

**Table 3:** Number and rate of non-motor symptoms (according to the results of MDS-UPDRS and NMSS scale).

	**Symptoms**	**PD group overall case (ratio %)**	**Early PD group overall case (ratio %)**	**Late PD group overall case (ratio %)**
	Overall case (ratio %)	37	24 (64.9)	13 (35.1)
1	Depression	31 (83.8)	20 (83.3)	11 (84.6)
2	Fatigue	28 (75.7)	18 (75.0)	10 (76.9)
3	Sleep disturbance	26 (70.2)	16 (66.7)	10 (76.9)
4	Anxiety	26 (70.2)	17 (70.8)	9 (69.2)
5	Gastrointestinal dysfunction	25 (67.6)	15 (62.5)	10 (76.9)
6	Bladder dysfunction	25 (67.6)	14 (58.3)	11 (84.6)
7	Apathy	19 (51.3)	11 (45.8)	8 (61.5)
8	Pain	17 (45.9)	8 (33.3)	9 (69.2)
9	Cognitive decline	13 (35.1)	7 (29.1)	6 (46.1)
10	Hallucination	2 (5.4)	2 (8.3)	0 (0.0)

### Comparison of Metabolites Between NMS Subgroups

Non-motor symptoms were categorized into three subgroups according to sleep condition, bowel condition, and cognitive state (as shown in Table 4). Cho/Cr in the right hippocampus in sleep disorder group was higher than that in the non-sleep disorder group (*t* = 2.184, *p* < 0.005). The mI/Cr in the right parahippocampal gyrus in gastrointestinal dysfunction group was lower than that in the non-gastrointestinal dysfunction group (*t* = −2.210, *p* < 0.005).

**Table 4:** The results of MRS in left/right cerebral hemisphere among the three subgroups of NMS-PD.

		**Sleep disorder group/non sleep disorder**	**Gastrointestinal dysfunction/non gastrointestinal dysfunction**	**Cognitive decline/non cognitive decline**
Substantia nigra	Left			Cr ↑; LL/Cr ↑
	Right			Cr ↑
Pallidum	Left			
	Right			Cr ↑
Thalamus	Left			Cr ↑; Cho/Cr ↑; LL/Cr ↑; mI/Cr↑
	Right			LL/Cr ↑; mI/Cr↑
Prefrontal cortex	Left			Cho/Cr ↑
	Right			
Hippocampus	Left			
	Right	Cho/Cr ↑		
Parahippocampal gyrus	Left			
	Right		mI/Cr ↓	mI/Cr ↑

**Figure 4:**
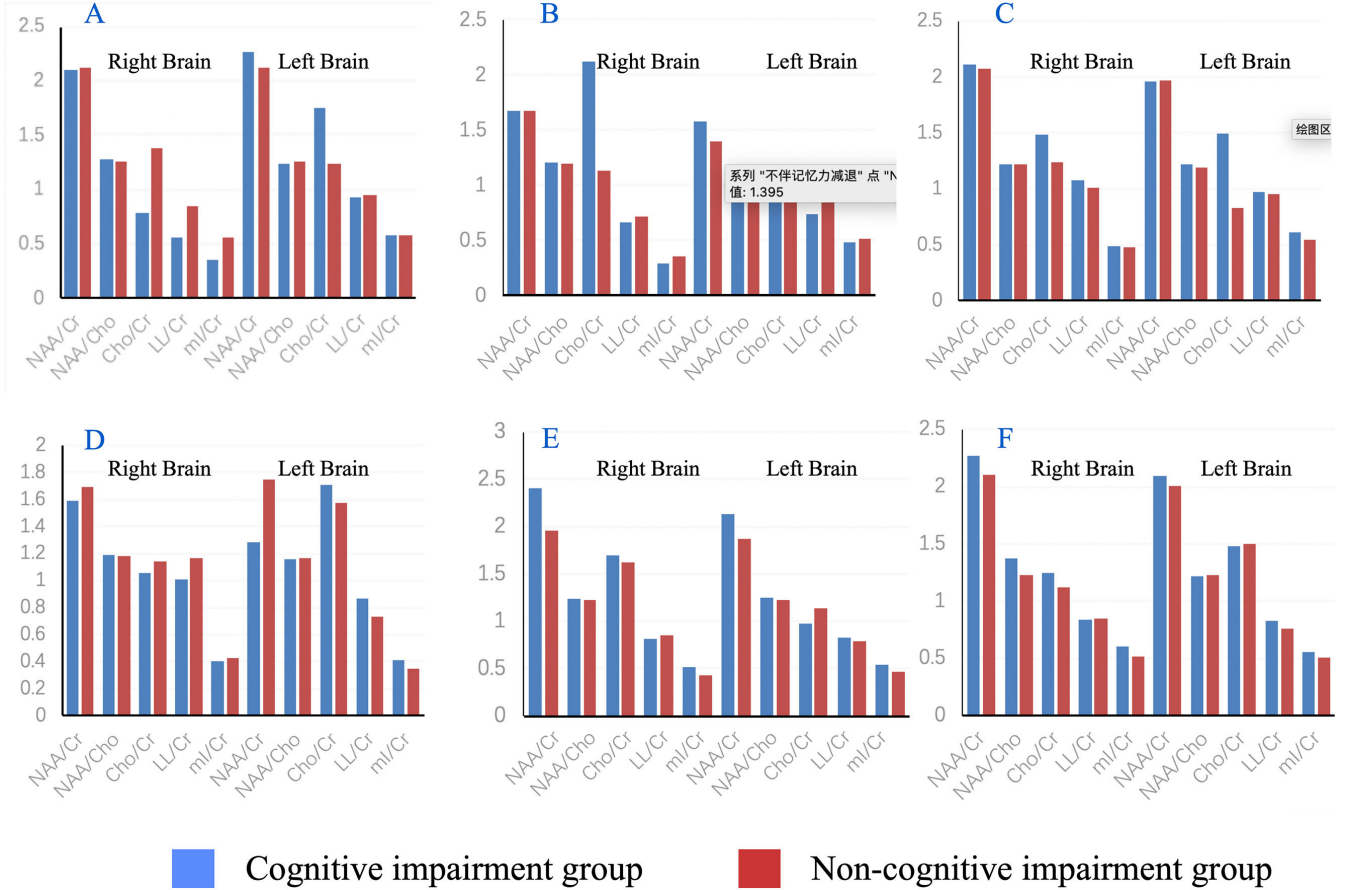
Histogram of mean metabolite results in substantia nigra **(A)**, pallidum **(B)**, thalamus **(C)**, prefrontal cortex **(D)**, hippocampus **(E)** and parahippocampal gyrus **(F)** with comparison between the cognitive impairment group and non-memory impairment group.

Comparing the cognitive group with non-cognitive group, four metabolites in twelve brain sites showed statistically difference (Cr, Cho/Cr, LL/Cr, mI/Cr, bilateral substantia nigra, bilateral globus pallidus, bilateral thalami, left prefrontal cortex, and right hippocampus), shown in Figure 4: (1) Cr in cognitive decline group was higher than non-cognitive declined group in bilateral substantia nigra (left: *t* = 2.575, *p* < 0.05, right: *t* = 4.701, *p* < 0.01), in the right pallidum (*t* = 2.245, *p* < 0.05), and in the left thalamus (*t* = 2.442, *p* < 0.05). (2) Cho/Cr in cognitive decline group was higher than non-cognitive declined group in the left thalamus (*t* = 2.467, *p* < 0.05) and the left prefrontal cortex (*t* = −2.142, *p* < 0.05). (3) LL/Cr in cognitive decline group was higher than non-cognitive declined group in left substantia nigra (*t* = 2.766, *p* < 0.05) and bilateral thalamus (*t* = 2.746 for left, 3.561 for right, *p* < 0.05). (4) mI/Cr in cognitive decline group was higher than non-cognitive declined group in bilateral thalamus (*t* = 2.184 for left, 2.096 for right, *p* < 0.05) and in right parahippocampal gyrus (*t* = 3.147, *p* < 0.05). Notably, other metabolites such as NAA/Cr and NAA/Cho had almost no statistical difference in all brain region in NMS subgroups (*p* > 0.05). Moreover, there was no statistically significant difference in Age and Score of H&Y, MDS-UPDRS, NMSS among the three subgroups. For more detail please see the Supplementary Table 2.

### Correlation Analysis Between Cerebral Metabolites and Clinical Factors of PD

Spearman's correlation analysis was used to learn the relationship between cerebral metabolites and clinical factors such as age, gender, disease duration, H&Y scale, UPDRS score, NMSS score, hypertension, and diabetes in patients with PD (as shown in Table 5). Regarding to the correlation of age and metabolites in patients with PD, positive correlation between age and Cr was observed in bilateral substantia nigra (*r* = 0.413 for left, 0.420 for right, *p* < 0.05), in bilateral pallidum (*r* = 0.356 for left side, 0.360 for right, *p* < 0.05), in left prefrontal cortex (*r* = 0.337, *p* < 0.05), in right hippocampus (*r* = 0.387, *p* < 0.05), and in left parahippocampal gyrus (*r* = 0.338, *p* < 0.05). For the correlation of gender and MRS in PD, NAA/Cr in the right substantia nigra or thalamus, NAA/Cho in the right parahippocampal gyrus were found to be positively correlated with men.

**Table 5:** Relationship between cerebral metabolites and clinical factors including age, gender, disease duration, H&Y scale, UPDRS score, NMSS score, hypertension and diabetes in PD patients was shown by using spearman correlation analysis.

**Position**	**Metabolites**	**Gender**		**Age**		**Disease duration**	
		***r***	***p***	***r***	***p***	***r***	***p***
Left substantia nigra	Cr	0.036	0.832	0.413^*^	0.011	−0.175	0.301
	NAA/Cr	0.250	0.135	0.069	0.685	0.176	0.296
	NAA/Cho	0.106	0.533	−0.018	0.917	0.092	0.589
	Cho/Cr	0.168	0.321	0.227	0.177	0.164	0.333
	LL/Cr	0.072	0.671	0.078	0.646	0.106	0.534
	mI/Cr	−0.013	0.940	−0.021	0.903	−0.343^*^	0.037
Right substantia nigra	Cr	0.129	0.447	0.420**	0.010	−0.073	0.669
	NAA/Cr	0.570**	0.000	0.248	0.139	−0.051	0.766
	NAA/Cho	0.279	0.095	0.260	0.121	−0.131	0.440
	Cho/Cr	0.121	0.475	−0.064	0.707	0.087	0.607
	LL/Cr	0.013	0.940	−0.194	0.251	0.317	0.056
	mI/Cr	−0.129	0.447	0.053	0.757	−0.188	0.265
Left pallidum	Cr	0.124	0.466	0.356^*^	0.030	0.074	0.664
	NAA/Cr	−0.155	0.360	0.171	0.312	0.154	0.364
	NAA/Cho	−0.008	0.964	0.171	0.311	0.141	0.406
	Cho/Cr	0.175	0.299	0.059	0.731	−0.014	0.936
	LL/Cr	0.000	1.000	−0.122	0.470	−0.062	0.715
	mI/Cr	0.052	0.762	0.040	0.816	0.002	0.990
Right pallidum	Cr	−0.077	0.649	0.360^*^	0.029	−0.132	0.437
	NAA/Cr	−0.139	0.411	0.024	0.890	−0.089	0.602
	NAA/Cho	−0.067	0.693	0.180	0.286	0.004	0.982
	Cho/Cr	0.309	0.062	−0.097	0.568	0.264	0.115
	LL/Cr	−0.026	0.880	−0.067	0.692	0.216	0.199
	mI/Cr	0.057	0.739	−0.311	0.061	−0.056	0.741
Left thalamus	Cr	0.222	0.187	0.285	0.087	0.106	0.533
	NAA/Cr	0.119	0.484	−0.005	0.977	−0.092	0.590
	NAA/Cho	−0.021	0.904	0.179	0.289	−0.031	0.854
	Cho/Cr	0.142	0.402	−0.129	0.445	0.088	0.605
	LL/Cr	−0.005	0.976	−0.043	0.802	0.245	0.144
	mI/Cr	0.015	0.928	0.137	0.418	−0.011	0.947
Right thalamus	Cr	0.031	0.856	0.221	0.188	−0.111	0.514
	NAA/Cr	0.389^*^	0.017	−0.047	0.780	0.095	0.576
	NAA/Cho	0.170	0.314	0.128	0.449	0.008	0.964
	Cho/Cr	−0.059	0.727	−0.015	0.930	0.203	0.229
	LL/Cr	0.224	0.182	0.128	0.451	0.092	0.589
	mI/Cr	−0.026	0.880	−0.020	0.908	0.037	0.828
Left prefrontal cortex	Cr	0.180	0.285	0.337^*^	0.041	0.096	0.570
	NAA/Cr	0.206	0.221	−0.260	0.120	0.222	0.186
	NAA/Cho	0.201	0.233	−0.080	0.637	0.103	0.545
	Cho/Cr	−0.134	0.429	0.140	0.410	−0.373^*^	0.023
	LL/Cr	0.075	0.660	0.012	0.946	−0.130	0.442
	mI/Cr	0.227	0.177	0.207	0.219	0.169	0.318
Right prefrontal cortex	Cr	−0.052	0.762	0.139	0.411	0.011	0.951
	NAA/Cr	0.013	0.940	0.044	0.794	−0.304	0.067
	NAA/Cho	0.070	0.682	0.012	0.945	−0.083	0.627
	Cho/Cr	−0.018	0.916	−0.079	0.643	0.224	0.183
	LL/Cr	−0.108	0.524	0.053	0.755	−0.097	0.567
	mI/Cr	0.077	0.649	−0.103	0.546	−0.029	0.864
Left hippocampus	Cr	−0.010	0.952	0.157	0.354	0.095	0.576
	NAA/Cr	−0.124	0.466	0.048	0.777	−0.223	0.185
	NAA/Cho	0.083	0.627	−0.080	0.637	0.057	0.740
	Cho/Cr	0.124	0.465	−0.322	0.052	−0.168	0.320
	LL/Cr	0.232	0.167	−0.051	0.765	0.140	0.408
	mI/Cr	−0.113	0.504	−0.148	0.381	−0.063	0.711
Right hippocampus	Cr	0.067	0.693	0.387^*^	0.018	−0.123	0.469
	NAA/Cr	−0.139	0.411	−0.032	0.849	0.011	0.949
	NAA/Cho	−0.183	0.278	−0.235	0.162	0.248	0.139
	Cho/Cr	0.085	0.617	0.027	0.876	0.152	0.368
	LL/Cr	−0.064	0.705	−0.314	0.058	0.172	0.309
	mI/Cr	−0.031	0.856	−0.122	0.472	−0.247	0.140
Left parahippocampal gyrus	Cr	0.005	0.976	0.338^*^	0.041	0.160	0.344
	NAA/Cr	−0.170	0.314	0.101	0.553	0.229	0.173
	NAA/Cho	−0.021	0.904	0.158	0.350	−0.128	0.450
	Cho/Cr	−0.286	0.086	0.216	0.198	0.224	0.183
	LL/Cr	0.103	0.544	0.013	0.941	0.077	0.649
	mI/Cr	−0.005	0.976	0.194	0.249	−0.154	0.362
Left parahippocampal gyrus	Cr	−0.113	0.504	0.056	0.741	−0.095	0.575
	NAA/Cr	−0.183	0.278	0.182	0.280	0.290	0.082
	NAA/Cho	0.387^*^	0.018	−0.178	0.292	0.287	0.085
	Cho/Cr	−0.508**	0.001	−0.099	0.561	0.020	0.906
	LL/Cr	−0.021	0.904	−0.069	0.685	0.089	0.600
	mI/Cr	0.103	0.544	−0.153	0.367	−0.136	0.423
**Position**	**Metabolites**	**H-Y classification**		**MDS-UPDRS scale score**		**NMSS scale score**	
		***r***	***p***	***r***	***p***	***r***	***p***
Left substantia nigra	Cr	0.089	0.602	0.208	0.217	0.081	0.634
	NAA/Cr	0.177	0.294	0.233	0.164	0.225	0.181
	NAA/Cho	−0.145	0.393	−0.131	0.439	0.028	0.870
	Cho/Cr	0.079	0.641	0.197	0.242	0.140	0.409
	LL/Cr	0.171	0.312	0.219	0.193	0.212	0.207
	mI/Cr	−0.236	0.160	−0.034	0.841	−0.192	0.256
Right substantia nigra	Cr	0.240	0.152	0.281	0.092	0.208	0.216
	NAA/Cr	−0.142	0.403	−0.083	0.624	0.067	0.694
	NAA/Cho	−0.059	0.728	0.144	0.395	0.197	0.243
	Cho/Cr	−0.193	0.25	0.405^*^	0.013	−0.339	0.051
	LL/Cr	−0.184	0.275	0.013	0.937	−0.201	0.234
	mI/Cr	−0.257	0.125	−0.044	0.796	−0.161	0.343
Left pallidum	Cr	0.041	0.81	0.106	0.534	−0.028	0.869
	NAA/Cr	0.110	0.517	0.073	0.666	0.080	0.637
	NAA/Cho	0.045	0.791	0.174	0.304	0.132	0.437
	Cho/Cr	0.165	0.328	−0.160	0.345	−0.215	0.200
	LL/Cr	0.011	0.947	0.374^*^	0.023	0.246	0.142
	mI/Cr	0.019	0.913	0.242	0.150	−0.056	0.743
Right pallidum	Cr	0.041	0.810	0.106	0.534	−0.028	0.869
	NAA/Cr	0.110	0.517	0.073	0.666	0.080	0.637
	NAA/Cho	0.045	0.791	0.174	0.304	0.132	0.437
	Cho/Cr	0.165	0.328	−0.160	0.345	−0.215	0.200
	LL/Cr	0.011	0.947	0.374^*^	0.023	0.246	0.142
	mI/Cr	0.019	0.913	0.242	0.150	−0.056	0.743
Left thalamu	Cr	−0.058	0.735	0.122	0.472	−0.021	0.904
	NAA/Cr	−0.036	0.830	0.115	0.496	0.116	0.495
	NAA/Cho	0.286	0.086	0.223	0.184	0.206	0.222
	Cho/Cr	0.213	0.207	0.639**	0.000	0.506**	0.001
	LL/Cr	0.163	0.334	0.338^*^	0.041	0.206	0.221
	mI/Cr	−0.233	0.164	0.075	0.660	−0.090	0.597
Right thalamus	Cr	−0.136	0.421	−0.011	0.947	0.007	0.968
	NAA/Cr	0.168	0.321	0.246	0.142	0.060	0.723
	NAA/Cho	0.106	0.531	0.138	0.416	0.022	0.897
	Cho/Cr	0.162	0.337	0.258	0.124	0.205	0.223
	LL/Cr	−0.024	0.887	0.123	0.470	0.108	0.525
	mI/Cr	0.049	0.772	0.133	0.433	−0.017	0.921
Left prefrontal cortex	Cr	0.146	0.389	0.253	0.130	0.119	0.484
	NAA/Cr	0.046	0.786	−0.009	0.956	0.052	0.762
	NAA/Cho	0.168	0.319	−0.021	0.903	0.165	0.329
	Cho/Cr	−0.309	0.063	−0.130	0.444	−0.280	0.093
	LL/Cr	0.077	0.651	0.139	0.411	0.081	0.635
	mI/Cr	−0.077	0.652	0.127	0.453	0.034	0.841
Right prefrontal cortex	Cr	0.107	0.529	0.160	0.345	0.097	0.569
	NAA/Cr	−0.265	0.113	−0.338^*^	0.041	−0.249	0.138
	NAA/Cho	−0.112	0.510	0.013	0.940	−0.072	0.672
	Cho/Cr	0.092	0.590	0.130	0.442	0.243	0.147
	LL/Cr	0.078	0.646	−0.033	0.847	−0.007	0.968
	mI/Cr	0.035	0.838	0.101	0.552	−0.007	0.968
Left hippocampus	Cr	−0.176	0.299	−0.049	0.774	–.354^*^	0.031
	NAA/Cr	0.145	0.391	0.135	0.426	0.296	0.075
	NAA/Cho	0.000	0.999	0.159	0.348	0.059	0.729
	Cho/Cr	−0.260	0.120	0.047	0.783	0.010	0.951
	LL/Cr	−0.164	0.332	−0.052	0.758	−0.085	0.615
	mI/Cr	−0.031	0.854	0.251	0.135	0.170	0.314
Right hippocampus	Cr	0.073	0.667	0.05	0.77	−0.083	0.627
	NAA/Cr	0.123	0.467	0.276	0.098	0.350^*^	0.034
	NAA/Cho	0.119	0.482	0.150	0.374	0.196	0.246
	Cho/Cr	−0.004	0.983	0.180	0.286	0.161	0.341
	LL/Cr	0.308	0.063	0.380^*^	0.020	0.121	0.477
	mI/Cr	−0.026	0.881	0.219	0.193	0.002	0.988
Left parahippocampal gyrus	Cr	0.068	0.689	0.160	0.345	−0.049	0.773
	NAA/Cr	0.270	0.106	−0.009	0.956	0.084	0.619
	NAA/Cho	0.179	0.288	0.024	0.886	0.119	0.483
	Cho/Cr	0.011	0.948	0.170	0.316	0.187	0.267
	LL/Cr	−0.133	0.432	0.091	0.592	−0.008	0.961
	mI/Cr	−0.064	0.705	0.072	0.670	0.075	0.657
Right parahippocampal gyrus	Cr	−0.029	0.865	0.305	0.067	0.128	0.451
	NAA/Cr	0.334^*^	0.043	0.237	0.158	0.163	0.335
	NAA/Cho	0.073	0.667	0.024	0.890	0.046	0.788
	Cho/Cr	−0.080	0.636	0.071	0.675	−0.110	0.517
	LL/Cr	0.123	0.470	0.297	0.075	0.142	0.402
	mI/Cr	−0.277	0.097	−0.017	0.922	−0.096	0.572
**Position**	**Metabolites**	**Hypertension**		**Diabetes mellitus**		
		***r***	***p***	***r***	***p***	
Left substantia nigra	Cr	0.356^*^	0.030	0.236	0.159	
	NAA/Cr	0.234	0.163	0.175	0.299	
	NAA/Cho	0.154	0.362	0.094	0.580	
	Cho/Cr	0.279	0.094	0.163	0.335	
	LL/Cr	−0.160	0.345	−0.236	0.159	
	mI/Cr	−0.066	0.700	−0.114	0.501	
Right substantia nigra	Cr	0.353^*^	0.032	0.204	0.226	
	NAA/Cr	0.228	0.175	0.041	0.811	
	NAA/Cho	0.165	0.328	0.126	0.456	
	Cho/Cr	−0.239	0.153	−0.143	0.400	
	LL/Cr	−0.137	0.419	−0.245	0.145	
	mI/Cr	−0.091	0.591	−0.073	0.666	
Left pallidum	Cr	0.228	0.175	0.024	0.886	
	NAA/Cr	0.091	0.591	0.237	0.159	
	NAA/Cho	0.157	0.354	0.045	0.792	
	Cho/Cr	0.046	0.789	0.024	0.886	
	LL/Cr	−0.311	0.061	−0.408^*^	0.012	
	mI/Cr	−0.188	0.265	−0.261	0.119	
Right pallidum	Cr	0.427**	0.008	0.318	0.055	
	NAA/Cr	−0.097	0.568	0.139	0.413	
	NAA/Cho	0.131	0.439	0.143	0.400	
	Cho/Cr	0.057	0.738	−0.273	0.102	
	LL/Cr	−0.285	0.087	−0.098	0.565	
	mI/Cr	−0.217	0.198	−0.293	0.078	
Left thalamus	Cr	0.299	0.072	0.041	0.811	
	NAA/Cr	−0.017	0.920	−0.139	0.413	
	NAA/Cho	0.111	0.512	0.057	0.737	
	Cho/Cr	0.148	0.381	0.049	0.774	
	LL/Cr	−0.063	0.712	0.065	0.701	
	mI/Cr	−0.074	0.663	−0.090	0.598	
Right thalamus	Cr	0.302	0.069	0.041	0.811	
	NAA/Cr	0.257	0.125	0.065	0.701	
	NAA/Cho	0.259	0.121	0.122	0.471	
	Cho/Cr	0.074	0.663	0.041	0.811	
	LL/Cr	0.040	0.815	−0.147	0.386	
	mI/Cr	−0.040	0.815	−0.057	0.737	
Left prefrontal cortex	Cr	0.228	0.175	−0.033	0.848	
	NAA/Cr	0.048	0.776	0.139	0.413	
	NAA/Cho	0.051	0.763	0.045	0.792	
	Cho/Cr	0.037	0.828	−0.033	0.848	
	LL/Cr	0.020	0.907	0.041	0.811	
	mI/Cr	−0.051	0.763	−0.179	0.288	
Right prefrontal cortex	Cr	0.239	0.154	0.049	0.774	
	NAA/Cr	−0.023	0.893	−0.041	0.811	
	NAA/Cho	0.160	0.345	−0.020	0.905	
	Cho/Cr	−0.011	0.947	0.037	0.829	
	LL/Cr	−0.040	0.815	−0.098	0.565	
	mI/Cr	−0.057	0.738	−0.041	0.811	
Left hippocampus	Cr	0.063	0.712	−0.057	0.737	
	NAA/Cr	−0.023	0.893	0.273	0.102	
	NAA/Cho	−0.063	0.712	−0.016	0.924	
	Cho/Cr	−0.168	0.320	−0.196	0.246	
	LL/Cr	−0.217	0.198	−0.342^*^	0.038	
	mI/Cr	−0.376^*^	0.022	−0.041	0.811	
Right hippocampus	Cr	0.171	0.312	0.139	0.413	
	NAA/Cr	0.251	0.134	0.342^*^	0.038	
	NAA/Cho	−0.126	0.459	0.188	0.266	
	Cho/Cr	0.182	0.280	−0.155	0.360	
	LL/Cr	−0.185	0.272	−0.110	0.517	
	mI/Cr	−0.251	0.134	−0.073	0.666	
Left parahippocampal gyrus	Cr	0.217	0.198	0.147	0.386	
	NAA/Cr	0.194	0.250	0.192	0.256	
	NAA/Cho	0.143	0.400	0.130	0.441	
	Cho/Cr	0.040	0.815	0.122	0.471	
	LL/Cr	−0.131	0.439	−0.187	0.266	
	mI/Cr	−0.251	0.134	−0.130	0.442	
Right parahippocampal gyrus	Cr	0.114	0.502	0.033	0.848	
	NAA/Cr	0.351^*^	0.033	0.330^*^	0.046	
	NAA/Cho	−0.011	0.947	−0.082	0.631	
	Cho/Cr	−0.091	0.591	−0.053	0.755	
	LL/Cr	0.017	0.920	0.049	0.774	
	mI/Cr	−0.120	0.480	−0.196	0.246	

*The relationship between metabolites and sex, age and disease cours*.

* *means P ≤ 0.05; n represents the number of cases*.

*The metabolites were correlated with H-Y classification, MDS-UPDRS scale score and NMSS scale score*.

* *means P ≤ 0.05; n represents the number of cases*.

*Association of metabolites with hypertension and diabetes mellitus*.

* *means P ≤ 0.05; n represents the number of cases*.

In terms of the correlation of UPDRS score and metabolites in patients with PD, LL/Cr were positively correlated with MDS-UPDRS in bilateral pallidum (*r* = 0.374 for both side, *p* < 0.05), in left thalamus (*r* = 0.338, *p* < 0.05) and right hippocampus (*r* = 0.380, *p* < 0.05). Cho/Cr was positively correlated in the right substantia nigra (*r* = 0.405, *p* < 0.05) and the left thalamus (*r* = 0.639, *p* < 0.05). NAA/Cr was statistically significant negative correlation in the right prefrontal gyrus (*r* = −0.338, *p* < 0.05). For the correlation of MMSS and metabolites in PD, there was statistically significant positive correlation between Cho/Cr in the left thalamus (*r* = 0.506), whereas negative correlation in the substantia nigra. Of note, there was no statistically correlation between metabolites and disease duration or H&Y scale (*p* > 0.05).

When analyzing the correlation between MRS and common chronic diseases (such as hypertension and diabetes) in patients with PD, positive correlations in hypertension were observed in Cr in bilateral substantia nigra (*r* = 0.356 for left, 0.353 for right) and in the right pallidum (*r* = 0.427), and identified in NAA/Cr in the right parahippocampal gyrus (*r* = 0.351). Similarly, positive correlations in diabetes were observed in NAA/Cr in the right parahippocampal gyrus and hippocampus (*r* = 0.330, 0.242, respectively). By contrast, negative correlation was found between hypertension and MRS in mI/Cr in the left hippocampus (*r* = −0.376), and between diabetes and MRS in LL/Cr in the left pallidum and hippocampus (*r* = −0.408 to −0.342, respectively).

## Discussion

We enrolled 48 participants (37 patients and 11 controls) and conducted the statistical difference between control group, the early stage of PD, and the late stage of PD in age, clinical characteristics. A total of 24 cases (H&Y: 1.29 ± 0.46) showed premotor symptoms and 13 cases (H&Y: 3.15 ± 0.38) showed progressive symptoms. Statistical difference was observed in Age, H&Y score, MDS-UPDRS score, and NMSS score among different groups (all *p* < 0.05). MRS was then performed in the specific cerebral regions, such as substantia nigra, pallidum, thalamus, prefrontal cortex, hippocampus, and parahippocampal gyrus, to investigate the change of neurometabolic in different stage of PD. There were no statistical differences between the early PD group and the control group with regard to the metabolites (*p* > 0.05), suggesting that no metabolic abnormalities were detectable in early stage patients with PD in our study, which is consistent with another study by Nora Weiduschat et al., who also used MRS but did not detect any effects of PD on the concentrations of NAA, Cho, and Cr in striatal and gray matter in early stage patients with PD (15). Of note, the olfactory center and the dorsal nucleus of the vagus oblongata, which were mainly affected by the lesions in the early stage of Braak's study (13), were not selected in the present and Nora's study. The failure to detect changes in early biomarkers may be related to the inadequate coverage of ROIs. However, some studies had pointed out that the neurodegenerative process can affect different brain regions in the early PD stage, not just affect local areas by dopaminergic denervation. Martin Klietz et al. utilized the MRS technique to estimate the changes in neurometabolic characteristics of early patients with PD in subcortical basal ganglia structures of bilateral frontal lobes, temporal lobes, parietal lobes, occipital lobes, and the cerebellum (16). Decreased NAA, Cho, and Cr were calculated in temporal-parietal, frontal, and occipital lobe. To put it in a nutshell, the alteration of pathology and neurometabolites in the present study may not spread to the substantia nigra, thalamus, hippocampus, and prefrontal lobes. Future studies should engage more ROIs for spectral analysis to identify more metabolic alterations and regions involved in the early pathological changes.

Compared late PD group with a control group or early group, higher Cr ratio and lower NAA/Cr ratio were observed in the late PD group (*p* < 0.05), which reflects the occurrence of neural cells apoptosis and compensatory for the neuronal energy consumption in certain regions. In addition, mI/Cr in the left thalamus in the late PD group was lower and suggested the activation and proliferation of glial cells in thalamic nuclei. In comparison to the early stage of PD, the disease has progressed and lesion-related metabolites were detected in the globus pallidus, thalamus, and hippocampus, which are consistent with the expansion of clinical symptom dimensions such as fatigue, abnormalities of sensation, and motor disorders. Notably, Cho/Cr and NAA/Cho had almost no statistically difference between the early PD group, the late PD group, and the control group (*p* > 0.05). The results in comparison with the late PD group and control group or early group suggested that higher Cr ratio, lower NAA/Cr, and lower mI/Cr might be potential biomarkers to imply the progression of PD.

Different severity and progression of neuropathology are determinants of Parkinson's symptoms (5). Pathology of the inclusion body was limited to the medulla oblongata/pontine tegmentum and olfactory bulb/anterior olfactory nucleus during pre-symptomatic stages 1–2, resulting in the obvious appearance of NMS, whereas relatively slight damage of motor symptoms, neural cells and glial cell disorder. In stages 3–4, the lesions gradually spread upward to the midbrain substantia nigra and nuclear grays, causing the gradual reduction of dopamine transmitters in caudate and putamen regions. Motor symptoms emerged at this point. In the end-stages 5–6, pathologies enter to the cerebral cortex and limbic system, which give rise to the manifestation of cerebral cortex dysfunction such as cognitive impairment and emotional disorders.

To find out the specific neurometabolites in NMS patients, we first counted the number of NMS in our included sample. More than 10 kinds of NMS occur in the early stage of patients with PD, mainly depression, anxiety, fatigue, and sleep disorders, and it gradually increases and worse as PD disease progresses. Specific markers presented in different cerebral regions were identified *via* comparing the alteration of neurochemicals between certain symptoms. The results of this study suggested that Cho/Cr in the right hippocampus in sleep disorder group was higher than that in the non-sleep disorder group (*t* = 2.184, *p* < 0.005). The increased level of Cho/Cr reflects the enhanced of membrane catabolism in the damaged site of hippocampus, which is consistent with a study showed the reduced of hippocampal volume in patients with insomnia (17). Of note, the underlying neuropathology of rapid eye movement (REM) sleep behavior disorder (RBD) is the degeneration of cholinergic neurons and neurons in brain stem and limbic system caused by aggregation of α-synuclein (18). Considering the high incidence of RBD (up to 65% of patients with PD), we speculated that sleep disturbances in patients may be related to RBD. Future study about the relationship between MRS metabolic results and RBD should be carried out.

A previously study conducted in patients with mild cognitive impairment or dementia, indicated a significant reductions of NAA in occipital region (19), dorsolateral prefrontal cortex and hippocampus (20), and a dramatically decreased NAA/Cr in temporoparietal cortex (21), occipital lobe (22), and posterior cingulate cortex (23). In contrast, the Cho/Cr ratio increased in the posterior cingulate in patient with mild cognitive impairment (22, 23). However, these statistical differences became no significant after adjusting for age, gender, and MDS-UPDRS, suggesting that MRS of posterior cingulate is not a useful biomarker for revealing the cognitive impairment in PD (23). In the present study, multiple metabolites in cognitive group were higher than that in non-cognitive group, such as Cr in bilateral substantia nigra, right pallidum, and left thalamus, Cho/Cr in the left thalamus and left prefrontal cortex, mI/Cr in bilateral thalamus and right parahippocampal gyrus, LL/Cr in left substantia nigra and bilateral thalamus. Together, pathologies associated with cognitive impairment were identified to gradually spread from midbrain to cerebral cortex. Cr, Cho/Cr, mI/Cr, and LL/Cr were all relatively high in the thalamus, suggesting that MRS of thalamus is sensitive for the detection of cognitive decline in PD. In addition, the alteration of neurochemicals in specific regions (such as Cr, Cho, MI, and LL) may be promising biomarkers to predict or identify cognitive decline in PD. Notably, other metabolites such as NAA/Cr and NAA/Cho had almost no statistical difference in the brain region in NMS subgroups (*p* > 0.05). There were no statistically differences between three NMS subgroups in Age, Score of H&Y, MDS-UPDRS, and NMSS.

Spearman's correlation analysis was used to study the connection between cerebral metabolites and clinical factors such as age, gender, disease duration, H&Y scale, UPDRS score, NMSS score, hypertension, and diabetes in patients with PD. Ratio of four metabolites, Cr, NAA/Cr, Cho/Cr, LL/Cr, mI/Cr, and NAA/Cho in twelve brain sites were obtained. Cr showed positive correlation with age. Normally, the decreased NAA was mainly associated with decreased gray matter volume and healthy neurons, while the increased Cho, Cr, and mI were related to the increased of white matter (24). The increased level of Cr is consistent with the reduced ratio of gray to white matter and the increased of energy metabolism requirements in the brain. On the other hand, for the relationship between metabolites and clinical symptoms, both Cho/Cr and LL/Cr had positive correlation with motor symptoms, while only Cho/Cr was positively correlated with the NMS. Choline is a component of cerebral myelin and cell membrane (16). The level of Cho might be a helpful hint to imply pathological changes in patients with PD.

## Limitation and Perspective

In the present study, it is difficult to acquire an accurate metabolic profile in the early stage of PD since some cerebral structures, which are too subtle to be detected and this neuroimaging technique may not be capable to avoid the partial volume effect of the adjacent structures. In addition, the metabolite concentrations of glutamate and GABA, as well as the region of the dorsal nucleus of the vagus oblongata and the olfactory center, were not measured due to a lack of appropriate processing software or equipment. Moreover, the sample size and the study time are not enough. The difference in the incidence of NMS between patients with PD and healthy individuals was not statistically analyzed. Further study could expand the sample size, extend the observation time, conduct more subgroups analysis, and engage more regions of interest (ROIs) for spectral analysis to identify more metabolic alteration. Currently, imaging techniques in the diagnosis of PD are still no clear. With the rapid development of MRS parameters, sequences, software and other technologies, the accuracy of MRS will continuously improve. Further study should be focused on the finding techniques which can directly reflect the changes of cell metabolites in different damaged sites so as to find out more objective markers that can be used to evaluate the site and severity of brain damage. Interestingly, the three-dimensional (3D) midbrain organoids, which are able to recapitulate features of the midbrain and simulate disease progression, can be combined with advanced neuroimaging techniques to investigate the complex pathological development.

## Conclusion

Proton magnetic resonance spectroscopy is an advanced tool to quantify neuronal integrity deficits and metabolic changes in PD. Although no symbolic biomarkers were detected in early patients with PD in our study, three biomarkers (Cr, NAA/Cr, and mI/Cr) experienced statistically significant differences, with an increased level of Cr, whereas reduced ratio of NAA/Cr and mI/Cr. In addition, NMS were classified into several subgroups (such as sleep disorder group, gastrointestinal dysfunction group, and cognitive impairment group). Cho/Cr might be a useful marker to identify sleep disorder, while decreased mI/Cr ratio might imply gastrointestinal dysfunction. MRS of thalamus is sensitive for the detection of cognitive decline in PD. The alteration of neurochemicals in specific regions (such as Cr, Cho, mI, and LL) may be promising biomarkers to predict or identify cognitive decline in PD. Furthermore, patients in the late stage of PD, or with cognitive impairment, have relatively extensive and severe brain lesions.

## Data Availability Statement

The raw data supporting the conclusions of this article will be made available by the authors, without undue reservation.

## Ethics Statement

The studies involving human participants were reviewed and approved by the Ethics Committee of the Second Affiliated Hospital of Shantou University Medical College and was performed according to the Declaration of Helsinki. The participants provided their written informed consent to participate in the study.

## Author Contributions

J-tG, XZ, L-fL, and S-yS contributed equally to study design and manuscript writing. Y-qG, Xl-Z, TZ, H-zW, J-qC, and Z-xY reviewed literature and analyzed data. X-hZ and T-xW conducted the neuroimaging. CW and Y-qZ assessed and treated the patients. All authors contributed to the article and approved the submitted version.

## Funding

This study was supported by the clinical research promotion project of Shantou University Medical College (201409), Guangdong Science and Technology Special Foundation (2021)88-27, and 2020 Li Ka Shing Foundation Cross-Disciplinary Research Grant (No. 2020LKSFG05D).

## Conflict of Interest

The authors declare that the research was conducted in the absence of any commercial or financial relationships that could be construed as a potential conflict of interest.

## Publisher's Note

All claims expressed in this article are solely those of the authors and do not necessarily represent those of their affiliated organizations, or those of the publisher, the editors and the reviewers. Any product that may be evaluated in this article, or claim that may be made by its manufacturer, is not guaranteed or endorsed by the publisher.
