# Supplementary material for: Proton Magnetic Resonance Spectroscopy for Diagnosis of Non-Motor Symptoms in Parkinson's Disease
Source: Front Neurol. 2022 Feb 28;13:594711. doi: 10.3389/fneur.2022.594711 (PMC8918562; doi:10.3389/fneur.2022.594711)
Supplement: Supplementary file 1 [file Table_1.docx]

Supplement Table 1. Cr, NAA/Cr, mI/Cr MRS results at different sites in the early, middle and late PD group and the control group (± s)

Creatine (Cr) MRS results

|  |  | Control group  （n=11） | Early PD group  （n=24） | Late PD group  （n=13） | *f* | *p* | *p1* | *p2* | *p3* |
| --- | --- | --- | --- | --- | --- | --- | --- | --- | --- |
| Substantia nigra | L | 71631±32616 | 54068±26728 | 58656±19048 | 1.672 | 0.199 | 0.395 | 0.075 | 0.617 |
|  | R | 82810±33117 | 63959±30665 | 81677±25015 | 2.243 | 0.118 | 0.575 | 0.090 | 0.092 |
| Globus pallidus | L | 53435±32706 | 33050±20873 | 52530±32347 | 3.215 | 0.050 | 0.562 | 0.055 | 0.043* |
|  | R | 62613±32187 | 50642±33219 | 54557±31959 | 0.507 | 0.606 | 0.550 | 0.319 | 0.729 |
| Thalamus | L | 56497±23535 | 37426±24474 | 63460±28868 | 5.027 | 0.011* | 0685 | 0.046 | 0.005* |
|  | R | 60877±25686 | 46181±24874 | 53826±27032 | 1.304 | 0.281 | 0.506 | 0.123 | 0.391 |
| Prefrontal cortex | L | 20013±13017 | 22863±21236 | 34464±21800 | 1.952 | 0.154 | 0.067 | 0.696 | 0.097 |
|  | R | 20710±9040 | 27089±16660 | 34895±22643 | 2.054 | 0.140 | 0.047* | 0.315 | 0.195 |
| Hippocampus | L | 74217±33838 | 69739±37075 | 69209±23326 | 0.085 | 0.919 | 0.714 | 0.713 | 0.963 |
|  | R | 74687±26213 | 64704±30232 | 71211±27571 | 0.522 | 0.597 | 0.946 | 0.344 | 0.513 |
| Parahippocampal gyrus | L | 65801±22403 | 50584±25719 | 66594±26320 | 2.319 | 0.110 | 0.995 | 0.104 | 0.071 |
|  | R | 62527±25796 | 58850±29254 | 57143±30417 | 0.108 | 0.898 | 0.843 | 0.728 | 0.864 |

*means *P* ≤0.05；n represents the number of cases；L means left; R means right; *p* is the value of *p* between the three groups, *p1* represents the *p* value of the control group and the late PD group, *p2* represents the *p* value of the control group and the early PD group, and *p3* represents the *p* value of the early PD group and the late group

N-acetylaspartate to creatine (NAA/Cr) MRS results

|  |  | Control group  （n=11） | Early PD group  （n=24） | Late PD group  （n=13） | *f* | *p* | *p1* | *p2* | *p3* |
| --- | --- | --- | --- | --- | --- | --- | --- | --- | --- |
| Substantia nigra | L | 1.746 ±0.428 | 2.064 ±0.523 | 1.322 ±0.525 | 3.900 | 0.027* | 0.030* | 0.089 | 0.144 |
|  | R | 1.615 ±0.744 | 2.109 ±0.477 | 1.106 ±0.264 | 4.003 | 0.025* | 0.004* | 0.100 | 0.988 |
| Globus pallidus | L | 1.101 ± 0.550 | 1.403 ±0.422 | 1.550 ±0.402 | 3.083 | 0.056 | 0.069 | 0.071 | 0.349 |
|  | R | 1.292 ± 0.517 | 1.573 ±0.466 | 1.859 ±0.763 | 2.961 | 0.062 | 0.059 | 0.183 | 0.152 |
| Thalamus | L | 1.604 ± 0.564 | 1.978 ±0.736 | 1.946 ±0.695 | 1.180 | 0.317 | 0.233 | 0.144 | 0.892 |
|  | R | 1.842 ± 0.560 | 1.973 ±0.504 | 1.304 ±0.865 | 1.805 | 0.176 | 0.081 | 0.573 | 0.135 |
| Prefrontal cortex | L | 1.705±1.294 | 1.614±1.107 | 1.573±0.590 | 0.049 | 0.952 | 0.293 | 0.813 | 0.910 |
|  | R | 1.865±0.984 | 1.799±0.715 | 1.405±0.439 | 1.576 | 0.218 | 0.129 | 0.805 | 0.122 |
| Hippocampus | L | 1.735 ±0.400 | 1.917 ±0.423 | 1.029 ±0.365 | 1.614 | 0.210 | 0.081 | 0.219 | 0.424 |
|  | R | 1.926 ±0.584 | 2.026 ±0.414 | 1.252 ±0.988 | 0.827 | 0.444 | 0.229 | 0.674 | 0.320 |
| Parahippocampal gyrus | L | 1.790 ±0.599 | 1.921 ±0.628 | 1.247 ±0.842 | 1.495 | 0.235 | 0.111 | 0.603 | 0.174 |
|  | R | 1.962 ±0.439 | 1.977 ±0.572 | 1.490 ±0.754 | 3.513 | 0.038* | 0.231 | 0.945 | 0.017* |

myo-Inositol to creatine (mI/Cr) MRS results

|  |  | Control group  （n=11） | Early PD group  （n=24） | Late PD group  （n=13） | *f* | *p* | *p1* | *p2* | *p3* |
| --- | --- | --- | --- | --- | --- | --- | --- | --- | --- |
| Substantia nigra | L | 0.618±0.536 | 0.594±0.422 | 0.527±0.389 | 1.233 | 0.301 | 0.197 | 0.186 | 0.892 |
|  | R | 0.733±0.590 | 0.519±0.285 | 0.385±0.175 | 0.955 | 0.392 | 0.272 | 0.184 | 0.852 |
| Globus pallidus | L | 0.462±0.348 | 0.780±0.612 | 0.267±0.180 | 0.403 | 0.671 | 0.146 | 0.866 | 0.059 |
|  | R | 0.329±0.222 | 0.349±0.235 | 0.350±0.159 | 3.369 | 0.043 | 0.752 | 0.494 | 0.728 |
| Thalamus | L | 0.633±0.478 | 0.776±0.447 | 0.446±0.450 | 3.590 | 0.036* | 0.428 | 0.422 | 0.012* |
|  | R | 0.703±0.545 | 0.527±0.359 | 0.458±0.223 | 4.46 | 0.170 | 0.373 | 0.754 | 0.964 |
| Prefrontal cortex | L | 0.649±0.559 | 0.567±0.413 | 0.383±0.184 | 2.004 | 0.147 | 0.959 | 0.993 | 0.944 |
|  | R | 0.499±0.543 | 0.452±0.252 | 0.414±0.235 | 0.246 | 0.783 | 0.235 | 0.534 | 0.447 |
| Hippocampus | L | 0.572±0.363 | 0.456±0.281 | 0.446±0.225 | 0.039 | 0.962 | 0.173 | 0.159 | 0.894 |
|  | R | 0.490±0.262 | 0.452±0.211 | 0.424±0.218 | 0.79 | 0.46 | 0.299 | 0.183 | 0.857 |
| Parahippocampal gyrus | L | 0.473±0.391 | 0.579±0.363 | 0.440±0.218 | 2.004 | 0.147 | 0.819 | 0.792 | 0.995 |
|  | R | 0.574±0.251 | 0.581±0.272 | 0.495±0.295 | 0.246 | 0.783 | 0.257 | 0.771 | 0.297 |
