# Supplementary material for: Proton Magnetic Resonance Spectroscopy for Diagnosis of Non-Motor Symptoms in Parkinson's Disease
Source: Front Neurol. 2022 Feb 28;13:594711. doi: 10.3389/fneur.2022.594711 (PMC8918562; doi:10.3389/fneur.2022.594711)
Supplement: Supplementary file 2 [file Table_2.docx]

Supplement Table 2. Comparison of metabolites in the Basal ganglia, Globus pallidus, Thalamus, Prefrontal cortex, Hippocampus, Parahippocampal gyrus region between cognitive impairment group and non-cognitive impairment group ( ± s)

Comparison of metabolites in the Basal ganglia region

| Group |  | Cr | NAA/Cr | NAA/Cho | Cho/Cr | LL/Cr | mI/Cr |
| --- | --- | --- | --- | --- | --- | --- | --- |
| Cognitive impairment | R | **96503±14774*** | 2.090±0.250 | 1.269±0.036 | 0.788±0.572 | 0.550±0.280 | 0.348±0.076 |
| Noncognitive impairment | R | **57552±26680*** | 2.117±0.473 | 1.252±0.073 | 1.380±1.424 | 0.832±0.437 | 0.558±0.346 |
| Cognitive impairment | L | **69393±20445*** | 2.256±0.514 | 1.239± 0.080 | 1.741±1.744 | **0.924±0.207*** | 0.576±0.330 |
| Noncognitive impairment | L | **49098±23297*** | 2.106±0.543 | 1.250±0.100 | 1.231±0.588 | **0.942±0.407*** | 0.565±0.326 |

*means *P* ≤0.05；n represents the number of cases；

Comparison of metabolites in the Globus pallidus region

| Group |  | Cr | NAA/Cr | NAA/Cho | Cho/Cr | LL/Cr | mI/Cr |
| --- | --- | --- | --- | --- | --- | --- | --- |
| Cognitive impairment | R | **71607±27339*** | 1.672±0.407 | 1.205±0.067 | 2.123±4.866 | 0.66±0.359 | 0.286±0.130 |
| Noncognitive impairment | R | **49031±29219*** | 1.674±0.672 | 1.194±0.096 | 1.126±0.792 | 0.719±0.350 | 0.348±0.233 |
| Cognitive impairment | L | 54606±19881 | 1.580±0.506 | 1.177±0.098 | 1.006±1.875 | 0.732±0.245 | 0.476±0.365 |
| Noncognitive impairment | L | 38132±28073 | 1.395±0.360 | 1.147±0.087 | 0.964±0.716 | 0.962±0.556 | 0.513±0.446 |

Comparison of metabolites in the Thalamus region

| Group |  | Cr | NAA/Cr | NAA/Cho | Cho/Cr | LL/Cr | mI/Cr |
| --- | --- | --- | --- | --- | --- | --- | --- |
| Cognitive impairment | R | **64919±19987*** | 2.111±0.424 | 1.223±0.079 | 1.487±0.840 | **1.074±0.302*** | **0.487±0.276*** |
| Noncognitive impairment | R | **44410±25520*** | 2.078±0.757 | 1.220±0.098 | 1.238±0.899 | **1.013±0.447*** | **0.474±0.277*** |
| Cognitive impairment | L | **64214±31848*** | 1.964±0.550 | 1.220±0.092 | **1.492±0.914*** | **0.971±0.251*** | **0.611±0.466*** |
| Noncognitive impairment | L | **43784±23719*** | 1.968±0.789 | 1.194±0.101 | **0.828±0.687*** | **0.954±0.533*** | **0.543±0.309*** |

Comparison of metabolites in the Prefrontal cortex region

| Group |  | Cr | NAA/Cr | NAA/Cho | Cho/Cr | LL/Cr | mI/Cr |
| --- | --- | --- | --- | --- | --- | --- | --- |
| Cognitive impairment | R | 38161±16893 | 1.589±0.254 | 1.187±0.074 | 1.056±0.930 | 1.009±0.611 | 0.403±.123 |
| Noncognitive impairment | R | 27928±17731 | 1.695±0.779 | 1.183±0.068 | 1.143±0.719 | 1.163±0.668 | 0.426±0.291 |
| Cognitive impairment | L | 38480±23806 | 1.285±0.359 | 1.159±0.069 | **1.711±0.531*** | 0.867±0.353 | 0.412±0.168 |
| Noncognitive impairment | L | 33351±26545 | 1.751±1.103 | 1.163±0.077 | **1.574±2.971*** | 0.734±0.412 | 0.351±0.269 |

Comparison of metabolites in the Hippocampus region

| Group |  | Cr | NAA/Cr | NAA/Cho | Cho/Cr | LL/Cr | mI/Cr |
| --- | --- | --- | --- | --- | --- | --- | --- |
| Cognitive impairment | R | 63112±23915 | 2.401±0.929 | 1.242±0.035 | 1.698±0.777 | 0.817±0.453 | 0.521±0.151 |
| Noncognitive impairment | R | 65110±32773 | 1.964±0.458 | 1.230±0.037 | 1.623±2.211 | 0.856±0.345 | 0.430±0.243 |
| Cognitive impairment | L | 62999±27402 | 2.128±0.262 | 1.250±0.031 | 0.973±0.578 | **0.827±0.410*** | 0.536±0.188 |
| Noncognitive impairment | L | 65212±37360 | 1.874±0.434 | 1.229±0.046 | 1.142±0.755 | **0.785±0.366*** | 0.465±0.314 |

Comparison of metabolites in the Parahippocampal gyrus region

| Group |  | Cr | NAA/Cr | NAA/Cho | Cho/Cr | LL/Cr | mI/Cr |
| --- | --- | --- | --- | --- | --- | --- | --- |
| Cognitive impairment | R | 71518±25673 | 2.269±0.875 | 1.370±0.472 | 1.247±1.037 | 0.832±0.408 | **0.602±0.157*** |
| Noncognitive impairment | R | 51883±29180 | 2.104±0.575 | 1.231±0.055 | 1.118±0.848 | 0.846±0.494 | **0.518±0.307*** |
| Cognitive impairment | L | 58183±26398 | 2.095±1.024 | 1.218±0.073 | 1.476±0.68 | 0.825±0.390 | 0.554±0.285 |
| Noncognitive impairment | L | 55263±27351 | 2.007±0.536 | 1.228±0.060 | 1.495±1.023 | 0.758±0.362 | 0.504±0.311 |
